# Local Structure Displacements and Electronic Structure of Sb-Substituted Rock-Salt Type AgBi_1−*x*_Sb_*x*_Se_0.8_S_0.6_Te_0.6_ System

**DOI:** 10.3390/ma18112578

**Published:** 2025-05-31

**Authors:** Lorenzo Tortora, Asato Seshita, Giovanni Tomassucci, Francesco Minati, Alina Skorynina, Laura Simonelli, Aichi Yamashita, Yoshikazu Mizuguchi, Naurang L. Saini

**Affiliations:** 1 Department of Physics, Sapienza University of Rome, P. le Aldo Moro 5, 00185 Rome, Italy; 2 Department of Physics, Tokyo Metropolitan University, 1-1 Minami-Osawa, Hachioji, Tokyo 192-0397, Japan; seshita-asato@ed.tmu.ac.jp (A.S.);; 3 CELLS—ALBA Synchrotron Radiation Facility, Carrer de la Llum 2-26, Cerdanyola del Valles, 08290 Barcelona, Spain

**Keywords:** thermoelectric materials, local structure, X-ray absorption spectroscopy, X-ray photoelectron spectroscopy

## Abstract

The cubic phase of the high-entropy alloy AgBi_1−*x*_Sb_*x*_Se_0.8_S_0.6_Te_0.6_ compound, characterized by the substitution of Sb for Bi in the structure to enhance phonon scattering, has been analyzed for local atomic displacements and electronic structure using a combination of X-ray absorption and X-ray photoelectron spectroscopy techniques. Notably, Ag K-edge and Bi L_3_-edge X-ray absorption measurements demonstrate a contraction of bond distances upon substitution due to the smaller size of Sb. Conversely, X-ray photoelectron spectroscopy reveals that, while Ag remains predominantly in the Ag^1+^ state across all samples, Bi and Sb exhibit a single valence state only for minimal Sb substitution. At higher Sb substitution levels, both Bi and Sb manifest mixed valence states, indicating complex electronic behavior that potentially influences the thermoelectric properties of the system. These findings suggest that optimizing the local structure through Sb substitution can be beneficial in enhancing the material’s thermoelectric performance.

## 1. Introduction

Following the new wave of investments in the field of clean energy and eco-friendly materials, thermoelectricity is experiencing significant momentum with the discovery of promising materials for practical applications [1,2,3,4,5,6]. The thermoelectric efficiency of a material is governed by the dimensionless figure of merit $ZT=\frac{S^{2}\sigma }{\kappa_{lat}+\kappa_{el}}$, where ZT≥1 is considered good enough for a thermoelectric (TE) material. Thermoelectric performance can thus be optimized in several ways: either by increasing electronic transport properties (i.e., the Seebeck’s constant *S* and the electric conductivity σ), or by reducing the lattice thermal conductivity ($\kappa_{lat}$). Since the latter approach is the most practical, given that *S* and σ are typically anti-correlated, much effort has been devoted to developing good TE materials by introducing point defects that act as phonon scatterers, thereby reducing $\kappa_{lat}$.

One of the most promising families of TE compounds is the ternary I-V-VI_2_ group, which includes AgBiSe_2_ [7,8,9]. These compounds are characterized by low thermal conductivity due to lattice distortions. Their high thermoelectric performance is commonly attributed to Ag/Bi anti-site disorder [10] and the activity of the Bi 6s lone pair [11,12,13], in particular at high temperatures in the cubic phase [14,15]. Recently, a stable room-temperature cubic phase of AgBiSe_2_ was stabilized through the substitution at the chalcogen (Ch) site in AgBiSe_2−2*x*_S_*x*_Te_*x*_ [16,17]. This high-entropy alloy concept [18,19,20] appears to yield promising thermoelectric properties in the mid-temperature range [16,17], thanks to reduced lattice thermal conductivity ($\kappa_{lat}$) resulting from increased configurational disorder and, consequently, more phonon scattering sites.

The same high-entropy approach has been employed to investigate the Sb substitution effects at the Bi sites, which are intrinsically susceptible to atomic deficiencies due to the Ag/Bi anti-site disorder. Apart from Sb being more environmentally friendly, the prominent lone-pair activity of Sb is anticipated to largely preserve the local distortions in the BiCh_6_ (Ch = Se, S, Te) octahedron. Furthermore, the substituted Sb is expected to introduce additional phonon scattering due to the enhanced configurational disorder. Indeed, AgSbCh_2_ compounds themselves exhibit high ZT [21,22,23,24,25,26,27]; however, unlike AgBiSe_2_, they are plagued by reliability issues due to the rapid degradation of their thermoelectric properties [28,29,30]. In contrast, the intercalation of Sb in high-entropy type cubic AgBi_1−*x*_Sb_*x*_Se_0.8_S_0.6_Te_0.6_ can be stabilized by a quenching method, resulting in stable and reproducible compounds [16,17,31].

X-ray absorption fine structure (XAFS) spectroscopy [32,33] is a specialized experimental technique that enables the investigation of the local structure and valence electronic structure of materials. Its widespread application has facilitated the study of thermoelectric materials, including AgBiSe_2_ based compounds [34]. Recently, we conducted a study of the local structure and electronic properties of high-entropy type AgBiSe_2*x*−2*x*_S_*x*_Te_*x*_ using Ag K-edge XAFS and XPS techniques [35]. This study revealed a gradual transformation in the local structure and geometry across the trigonal to cubic phase transition. Notably, the XPS results indicated an anomalous chemical potential shift, while both Bi and Ag remained in a single valence state throughout the Ch substitution process. In this work, we aim to investigate the local structure and electronic properties of the cubic phase of AgBiSe_2*x*−2*x*_S_*x*_Te_*x*_ compounds with Sb substitution. Ag K-edge and Bi L_3_-edge XAFS measurements, combined with XPS, are used to study the local atomic displacements and the electronic structure of AgBi_1−*x*_Sb_*x*_Se_0.8_S_0.6_Te_0.6_ in the substitution range of x = 0–0.6. Extended X-ray absorption fine structure (EXAFS) analysis reveals a contraction of Ag-Ch and Bi-Ch bond distances with increasing Sb content. Both X-ray absorption near edge structure (XANES) and XPS show that Ag remains in the Ag^1+^ state for all substitutions. Furthermore, XPS measurements indicate that Bi and Sb are in mixed valence states at higher substitution levels. A non-trivial electronic structure is observed for small Sb substitution, likely due to the presence of additional impurity levels in the band gap. The local distortions and electronic properties of the system are discussed in light of their thermoelectric properties. The results suggest pathways for manipulating AgBiCh_2_-based thermoelectric materials to optimize their properties for applications.

## 2. Materials and Methods

Polycrystalline samples of cubic Bi_1−*x*_Sb_*x*_Se_0.8_S_0.6_Te_0.6_, prepared via solid-state reaction, were fully characterized for their thermoelectric transport and average structural properties prior to the X-ray absorption and XPS measurements [16,17,31]. Ag K-edge and Bi L_3_-edge X-ray absorption measurements were carried out in transmission mode at the CLAESS beamline of the ALBA synchrotron radiation facility in Cerdanyola del Vallés (Barcelona, Spain) [36]. Powders of AgBi_1−*x*_Sb_*x*_Se_0.8_S_0.6_Te_0.6_ were mixed with an organic binder and pressed into pellets with a diameter of 13 mm. The thickness of the pellets and the sample quantity were optimized to ensure the X-ray absorption jump reaching almost unity. The incident intensity ($I_{0}$), the transmitted intensity through the sample ($I_{t}$), and the transmission through a reference sample were measured using ionization chambers. A double-crystal Si(311) monochromator, along with a Pt-coated focusing mirror to filter out higher harmonics, was used on the multipole wiggler beamline. The EXAFS oscillations were extracted from the X-ray absorption spectra using the standard procedure with ATHENA software [37]. Each sample underwent four to five absorption scans to ensure spectral reproducibility.

XPS measurements were performed using the in-house UHV system at the Sapienza University of Rome, equipped with a double-anode XR-50 X-ray source and a multi-channel AR125 Omicron electron analyzer (Berlin, Germany). The base pressure during the measurements was 1.2 × 10^−9^ mbar. The Al Kα anode (hν = 1486.6 eV) was used as the excitation source, with some selected samples also measured using a Mg Kα source (hν = 1253.6 eV). The incident and emission angles for the measurements were approximately 45°. Clean surfaces were obtained by in-situ scraping of sintered pellets using a diamond file. To calibrate the energy scale, repeated Au 4f core-level XPS measurements were performed on a clean gold foil. The C 1*s* core level on the sample manipulator served as an additional energy reference. Multiple scans were acquired for each core level to ensure a high signal-to-noise ratio. The X-ray photoelectron spectroscopy tool (XPST) extension in Igor Pro 9 [38], which permits one to perform detailed core-level peak analysis, was used to fit the XPS core level profiles.

## 3. Results and Discussion

### 3.1. The Local Structure Investigations by EXAFS Spectroscopy

**Figure 1:**
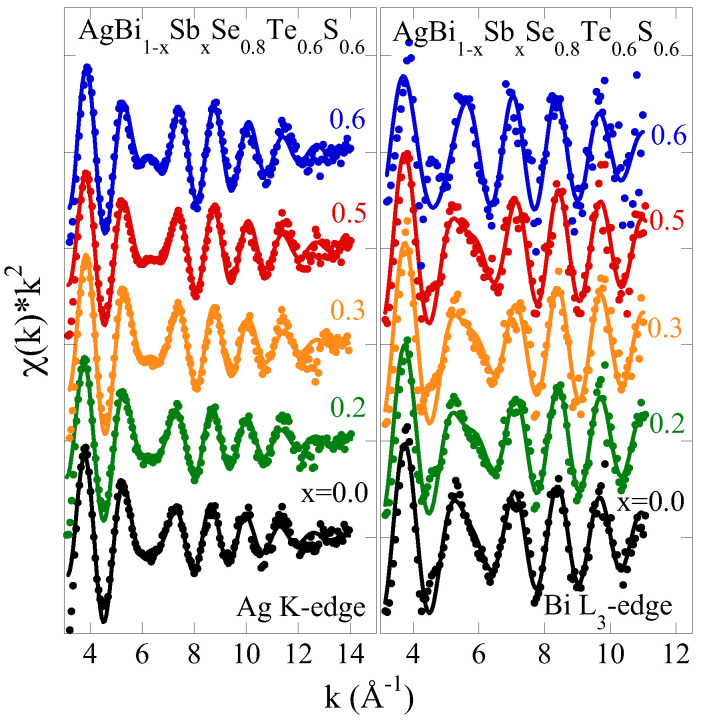
Ag K-edge (**left**) and Bi L_3_-edge (**right**) EXAFS oscillations for AgBi_1−*x*_Sb_*x*_Se_0.8_S_0.6_Te_0.6_ with variable Sb contents. The solid lines represent model fits (see text), including contributions of Ag-Ch (Ag K-edge) and Bi-Ch (Bi L_3_-edge) bond distances.

Figure 1 shows the Ag K-edge and Bi L_3_-edge EXAFS oscillations at 300 K for several Sb substitutions in AgBi_1−*x*_Sb_*x*_Se_0.8_S_0.6_Te_0.6_. The oscillations are multiplied by $k^{2}$ to enhance their visibility at higher *k*-values. The Ag K-edge and Bi L_3_-edge EXAFS oscillations are visible up to approximately 14 Å^−1^ and 11 Å^−1^ , respectively, revealing a small but noticeable effect of Sb substitution. These oscillations provide insight into the partial pair distribution function around the photoabsorbing Ag and Bi atoms in AgBi_1−*x*_ Sb_*x*_Se_0.8_S_0.6_Te_0.6_. The small variations in the EXAFS signals indicate that Sb substitution may have limited effect on the local structure; however, it still impacts the transport properties of AgBi_1−*x*_Sb_*x*_Se_0.8_S_0.6_Te_0.6_ substantially.

**Figure 2:**
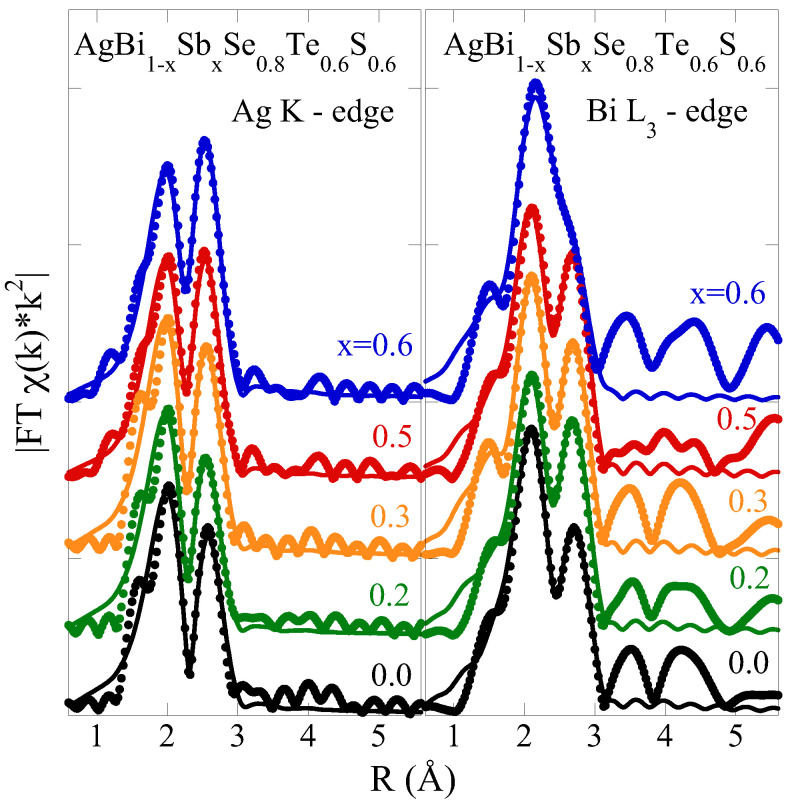
Fourier transform magnitudes of Ag K-edge (**left**) and Bi L_3_-edge (**right**) EXAFS oscillations (see Figure 1) for AgBi_1−*x*_Sb_*x*_Se_0.8_S_0.6_Te_0.6_ with variable Sb contents. The Fourier transforms are performed using Gaussian window with the *k*-ranges being 3–14 Å^−1^ and 3–11 Å^−1^, respectively, for the two edges. The solid lines represent model fits (see text).

The partial pair distribution can be represented in real space by the Fourier transform (FT) magnitude of the EXAFS oscillations, as displayed in Figure 2. The FT magnitudes of the EXAFS oscillations are obtained by convolving the k-space spectra with a Gaussian window over a *k*-range of 3–14 Å^−1^ for the Ag K-edge and 3–11 Å^−1^ for the Bi L_3_ edge. The FTs reveal peak structures between approximately 2 and 4 Å, which correspond to contributions from neighboring chalcogen (S, Se, and Te) atoms surrounding the Ag and Bi atoms in the structure. Contributions from the longer distances are expected beyond about 4 Å, along with multiple scatterings, which are largely suppressed. This kind of distribution is typical for systems with significant intrinsic disorder, such as AgBi_1−*x*_Sb_*x*_Se_0.8_S_0.6_Te_0.6_. It is noteworthy that the FT of the Bi L_3_-edge EXAFS for the x = 0.5 sample differs substantially from others beyond the main contribution (∼4 Å). Consequently, we cannot entirely exclude the possibility of the presence of a microscopic amorphous impurity phase containing Bi that was not detectable by diffraction. Nevertheless, the shape of the spectra is consistent with previously measured data on the Ag K-edge EXAFS of AgBiSe_2−2*x*_S_*x*_Te_*x*_ [35], showing that Sb has only a marginal effect on the local configuration.

The local structural parameters can be derived from the standard EXAFS analysis. The longer bonds contributions are suppressed and only the nearest chalcogen neighbors (Se, S, and Te) contributions are considered for the EXAFS model fits using the standard EXAFS equation [32,33] based on single scattering approximation:

(1) $$\chi \left(k\right)=\sum_{i}\frac{N_{i}S_{0}^{2}}{kR_{i}^{2}}f_{i}\left(k,R_{i}\right)e^{\frac{-2R_{i}}{\lambda }}e^{-2k^{2}\sigma_{i}^{2}}sin\left[2kR_{i}+\delta_{i}\left(k\right)\right]$$

where $N_{i}$ in Equation (1) is the number of atoms located at a distance $R_{i}$ from the photoabsorbing atoms (Ag or Bi) in the structure, while $\delta_{i}$ refers to the phase shifts, and $f_{i}$(k, $R_{i}$) is the backscattering amplitude. The EXAFS signal also depends on the mean free path of the photoelectron (λ) and the $\sigma_{i}^{2}$ parameter, known as the EXAFS Debye–Waller factor (DWF). The scale factor ($S_{0}^{2}$) accounts for the many-body effects that cause a loss of amplitude in the photoelectron propagation in material before being detected [32,33,39]. The initial model for the current analysis is based on the average structure of AgBiCh_2_ (Ag and Bi sites octahedrally coordinated with six chalcogen atoms: Se, S, and Te). The model employs three atomic shells to probe the surrounding chalcogen atoms around Ag and Bi. The number of near-neighbor chalcogen atoms ($N_{i}$) surrounding the octahedrally coordinated photoabsorbing atoms are kept constant, i.e., $N_{Se}$ = 2.4, $N_{S}$ = 1.8, and $N_{Te}$ = 1.8. The scale factor $S_{0}^{2}$ is set to 1. The only fit parameters in the model fits are bond distances ($R_{i}$) and corresponding DWFs describing the mean square relative displacement (MSRD) of the pair of atoms. Therefore, only six parameters are allowed to vary in the non-linear least-squares fits of the EXAFS data to Equation (1). The number of independent data points for the analysis is N_*ind*_ ≃ 15 for the Ag K-edge EXAFS and ≃11 for the Bi L_3_-edge EXAFS (N_*ind*_∼2ΔkΔR/π, where Δk and ΔR represent the fit ranges in k-space and R-space, respectively), much larger than the fit parameters. The model fits are presented as solid lines in Figure 1 and Figure 2, illustrating fits in *k*-space and R-space, respectively.

**Figure 3:**
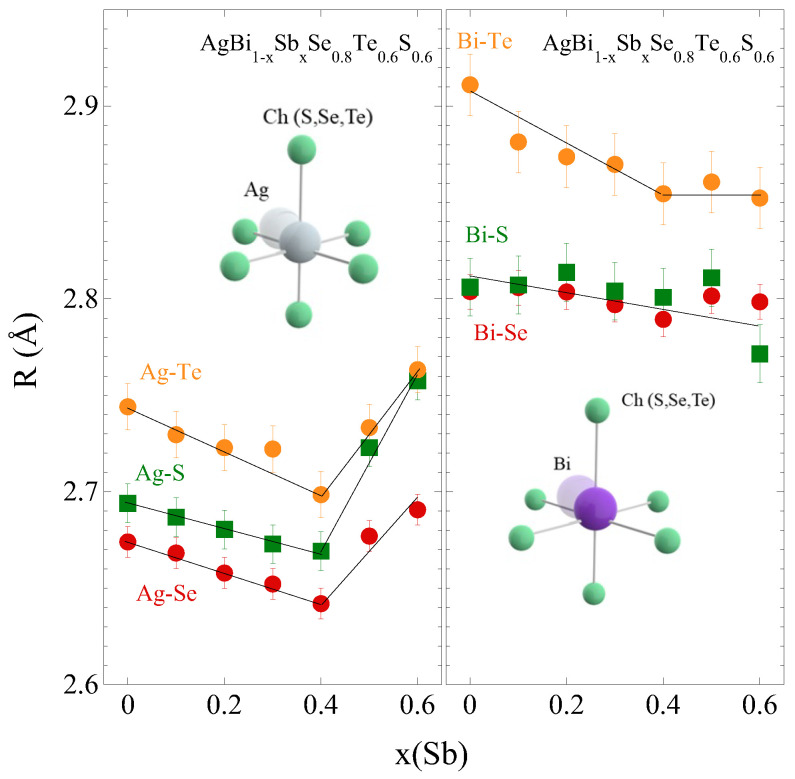
Evolution of near neighbor bond lengths from Ag (**left**) and Bi (**right**) atoms in AgBi_1−*x*_Sb_*x*_Se_0.8_S_0.6_Te_0.6_ structure. The local structure cartoons are included as insets showing octahedral coordinations of Ag and Bi. The error bars represent maximum error evaluated by standard EXAFS error analysis based on the parabola method.

Figure 3 illustrates the near-neighbor distances of the Ch atoms, determined by the EXAFS model fits. In the pristine sample (x = 0), the Ch atoms appear at almost similar distances from Ag, i.e., ∼2.67 Å (Se), ∼2.71 Å (S), and ∼2.73 Å (Te), consistent with the previous EXAFS measurements on a sample with similar stoichiometry [35]. Similarly, Se, S and Te distances from Bi for the pristine sample are ∼2.80 Å, ∼2.81 Å, and ∼2.91 Å, respectively. The three Ag-Ch distances show a slight shrinkage for x ≤ 0.4. The same trend is observed for Bi-Ch distance, indicating an overall contraction of the lattice, likely to be due to the smaller size of Sb. Beyond x = 0.4, the Ag-Ch distances appear to elongate, whereas the Bi-Ch distances hardly show any change.

**Figure 4:**
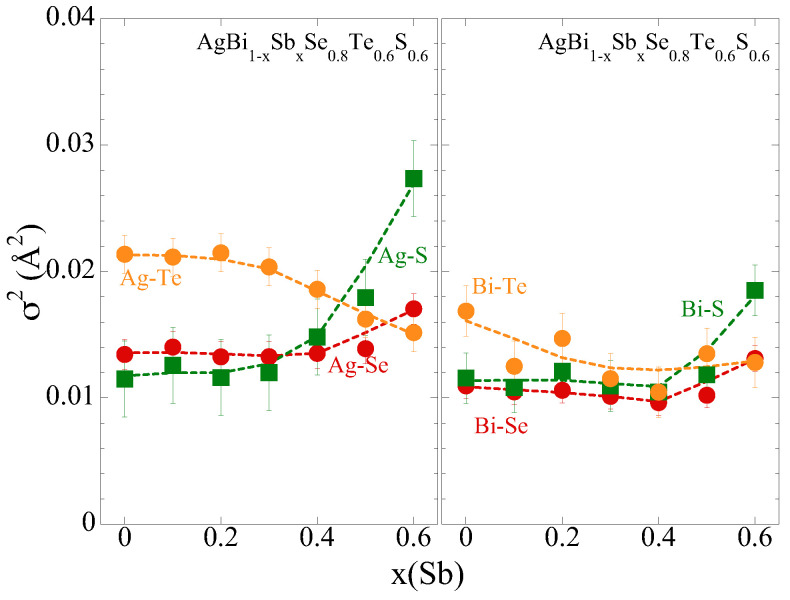
Debye–Waller factor parameter ($\sigma^{2}$) of Ag-Ch (**left**) and Bi-Ch (**right**) distances in AgBi_1−*x*_Sb_*x*_Se_0.8_S_0.6_Te_0.6_. The $\sigma^{2}$ is determined by the EXAFS model fits of Ag K-edge and Bi L_3_-edge EXAFS measured on samples with different Sb substitution.

The local structure changes can be further evaluated by the EXAFS DWFs of the pair of atoms. The $\sigma^{2}$ of Ag-Ch and Bi-Ch bondlengths can be seen in Figure 4. Again, $\sigma^{2}$ for x ≤ 0.4 does not show any significant change. Beyond x = 0.4, the $\sigma^{2}$ of Ag-Se and Ag-S distances tend to increase with a similar trend in $\sigma^{2}$ of Bi-Se and Bi-S. The increase in the $\sigma^{2}$ is due to increased configurational disorder. Incidentally, the $\sigma^{2}$ of Ag-Te reveals a decrease, while that of Bi-Te hardly showing any change. Therefore, it appears clear that the overall effect of Sb on the local structure is marginal. This could be attributed to the high mixing entropy of the system with robust mechanical characteristics. It is also clear that the local structural changes depend on the substitution content, i.e., a smaller effect at lower substitution levels and a larger effect at higher substitution levels. Further information on the local geometry can be obtained from XANES analysis.

### 3.2. The Local Geometry by XANES Spectroscopy

**Figure 5:**
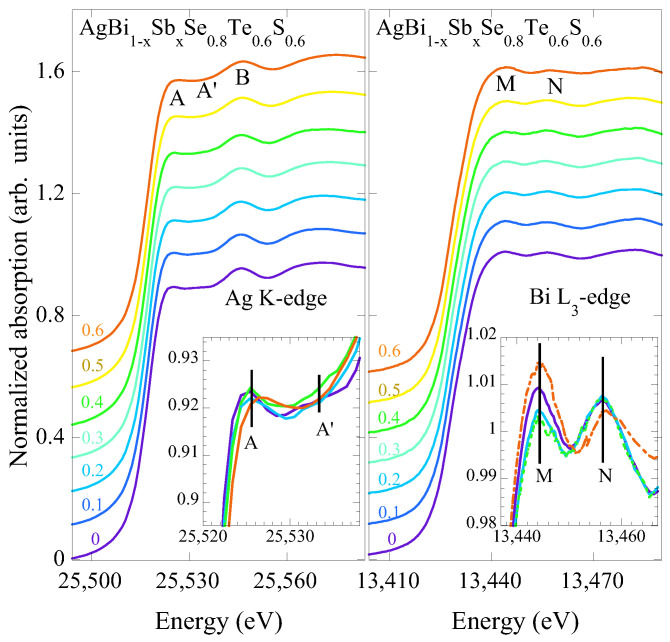
Ag K edge (**left**) and Bi L_3_-edge (**right**) XANES spectra for all the studied samples. The spectra are shifted vertically for better visualization. Insets are zoom-overs of the main features A, A’, and B in Ag K edge and M and N in Bi L_3_-edge XANES.

Unlike EXAFS, XANES spectroscopy probes higher-order atomic correlations, providing information on the local geometry and valence electronic states. XANES features are sensitive probes of the local geometry, in addition to providing important information on the unoccupied electronic states, making it a very useful tool to investigate complex systems such as AgBi_1−*x*_Sb_*x*_Se_0.8_S_0.6_Te_0.6_. Figure 5 shows the Ag K-edge and Bi L_3_-edge XANES spectra of AgBi_1−*x*_Sb_*x*_Se_0.8_S_0.6_Te_0.6_, normalized with respect to the atomic absorption, which was determined by fitting a linear function over a broad energy range away from the absorption edges. The main features in the Ag K-edge XANES are denoted as A (∼25,524 eV), A’ (∼25,533 eV), and B (∼25,546 eV). Similarly, the Bi L_3_-edge XANES features are denoted as M (∼13,444 eV) and N (∼13,456 eV). The insets show zoomed-in views of the main XANES features, highlighting the effect of Sb substitution on the local geometry and valence electronic structure.

The Ag K-edge XANES primarily involves Ag 1*s* to 5*p* dipole transitions. However, due to large core-hole lifetime broadening, a distinct white line transition is not observed [35]. There seems to be a small but non-negligible effect of Sb on the Ag K-edge XANES features. The Ag K-edge energy (∼25,517 eV) for AgBi_1−*x*_Sb_*x*_Se_0.8_S_0.6_Te_0.6_, hardly changing with Sb content and similar to the one for Ag_2_O, confirms that silver should be in the Ag^1+^ state. There is also a small but an apparent shift of features A towards higher energy (∼1 eV), consistent with a contraction of Ag-Ch bondlengths. The Bi L_3_-edge XANES probes transitions from the Bi 2*p* to the unoccupied Bi 6*d* and admixed Bi 6*p* states in the continuum, which are also admixed with the Bi 6*s* and Ch *p* states due to the presence of Bi^3+^ [40]. Therefore, the peaks M and N are sensitive to the local geometry around the Bi atom. The spectral differences in Bi L_3_-edge XANES are much larger than those observed in the Ag K-edge XANES. This is clear from the zoom over the peaks M and N, which reveals that peak M changes systematically with Sb substitution.

**Figure 6:**
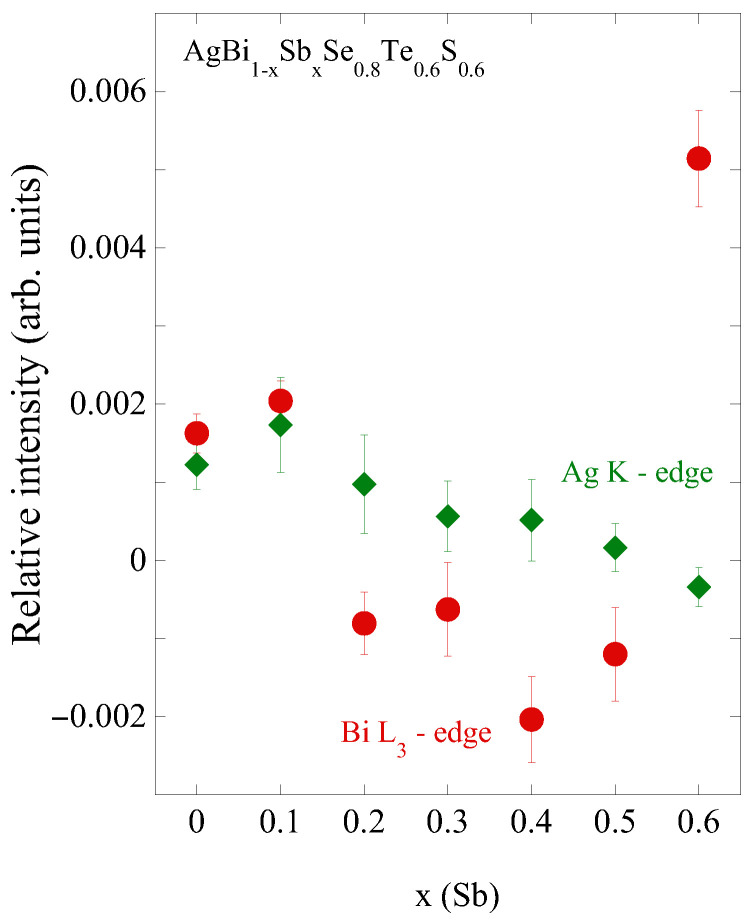
Relative spectral intensities of main XANES features with Sb substitution. The Bi L_3_-edge features (filled circles) change anomalously with Sb substitution, unlike the Ag K-edge features (filled diamonds) showing small but a gradual change.

To track the effect of Sb substitution on the local geometry, we have evaluated the relative intensities of peak A and A’ (I_*A*_ − I_*A*′_/I_*A*_ + I_*A*′_) in the Ag K-edge XANES and the realtive intensities of M and N (I_*M*_ − I_*N*_/I_*M*_ + I_*N*_) in Bi L_3_-edge XANES. Figure 6 shows the evaluated relative intensities with Sb substitution. Once again, it is clear that the main effect occurs around Bi, with an anomalous evolution of the local geometry as Sb being substituted, while the local geometry of Ag shows a smaller but gradual change. Considering the complexity of the system, it is difficult to provide a quantitative correlation between the local displacements and the relative spectral weight of XANES features. However, the observed evolution serves as a good marker of the changing local geometry with Sb substitution.

### 3.3. The Electronic Structure Studies by XPS

**Figure 7:**
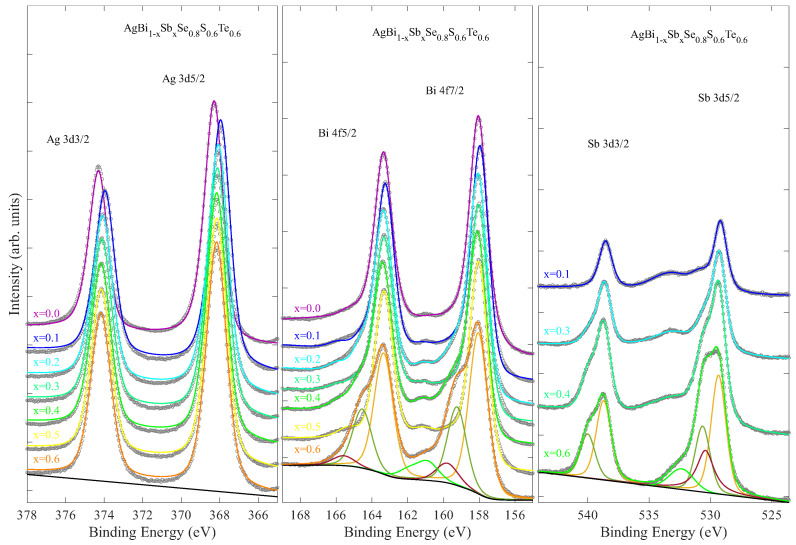
Ag 3*d* (**left**), Bi 4*f*, (**middle**) and Sb 3*d* (**right**) core-level XPS of AgBi_1−*x*_Sb_*x*_Se_0.8_S_0.6_Te_0.6_ for several Sb substitutions. The XPS profile fits are shown by solid lines. Different components are shown with the background function for the lowest spectrum.

**Figure 8:**
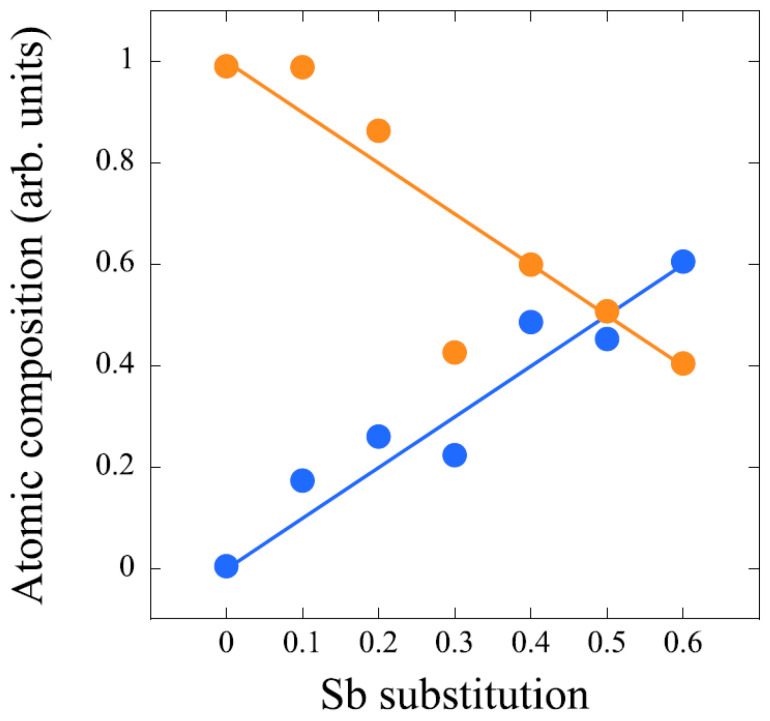
Measured Bi and Sb composition (dots) relative to their respective nominal values (solid lines).

XPS measurements were carried out on different core levels of AgBi_1−*x*_Sb_*x*_Se_0.8_S_0.6_Te_0.6_. Figure 7 shows Ag 3*d*, Bi 4*f*, and Sb 3*d* core-level XPS spectra for different Sb substitutions. First, the stoichiometry of the Bi/Sb was checked. Figure 8 shows the relative spectral weight of Bi and Sb that is close to the nominal values. Small deviations from the nominal values can be due to differences in the surface morphology illuminated by the X-ray beam for optimized photoelectron counts.

The binding energy of Ag 3$d_{5/2}$ is 368.16 eV for AgBiSe_0.8_S_0.6_Te_0.6_, which is similar to the value reported in the Ag 3*d* XPS of a different sample with the same stoichiometry [35]. Furthermore, the Ag 3*d* XPS is characterized by a single component, indicating that Ag remains in the Ag^1+^ state across Sb substitution. Similarly, the binding energy of Bi 4$f_{7/2}$ appears as a single component at 158.04 eV for the x = 0 sample, characteristic of the Bi^3+^ state. XPS profile fitting was used to obtain further information on the evolution of the core-level electronic structure with Sb substitution. The profile fits of the Ag 3*d* and Bi 4*f* core levels are included as solid lines in Figure 7. Within the energy resolution of approximately 1 eV, the Ag 3*d* XPS shows only a single component for all samples. On the other hand, the Bi 4*f* and Sb 3*d* core levels are characterized by one component for x ≤ 0.1 and two components beyond this, indicating a tendency toward mixed valency of both Bi and Sb in the Sb-substituted samples.

**Figure 9:**
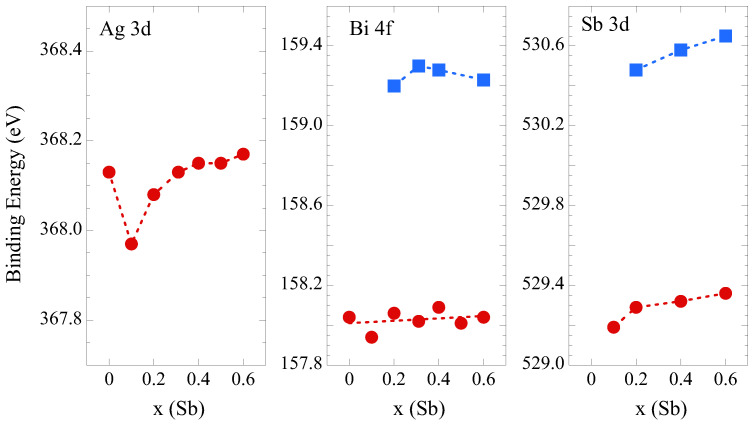
Ag 3*d* (**left**), Bi 4*f* (**middle**), and Sb 3*d* (**right**) core-level binding energies in AgBi_1−*x*_Sb_*x*_Se_0.8_S_0.6_Te_0.6_. The binding eneries are determined by core-level profile fits. The dashed line is a guide for the eyes.

Figure 9 shows the binding energies of Ag 3*d*, Bi 4*f*, and Sb 3*d* core levels. The Ag 3*d* binding energy is decreased by ∼0.2 eV for x = 0.1 and tends to show a small increase or remains constant with further substitutions. The Bi 4*f* and Sb 3*d* binding energies also show a non-negligible increase with the Sb substitution. This seems to be related with different mixing behavior of Sb in Bi_1−*x*_Sb_*x*_Se_0.8_S_0.6_Te_0.6_, i.e., an initial homogenous mixing for x = 0.1 sample followed by a local inhomogeneity with possible coexistance of Bi_1−*x*_Sb_*x*_Se_0.8_S_0.6_Te_0.6_ and AgSbCh_2_/Sb_2_Ch_3_. Indeed, previous studies on Sb/Bi intercalated compounds indicated tendency for Bi and Sb to have phase segregation [41,42].

**Figure 10:**
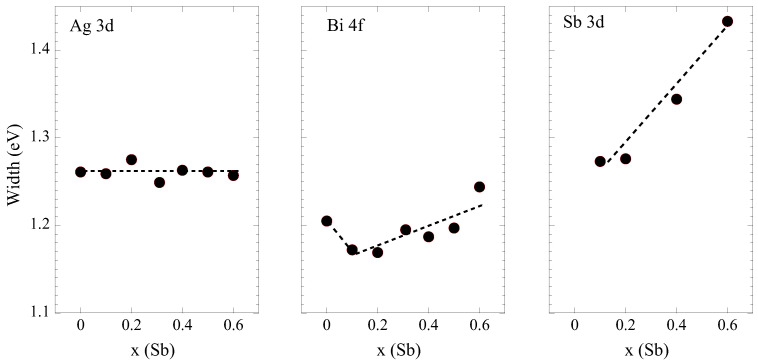
Core-level line widths for Ag 3*d* (**left**), Bi 4*f* (**middle**), and Sb 3*d* (**right**) in AgBi_1−*x*_Sb_*x*_Se_0.8_S_0.6_Te_0.6_. The dashed line is a guide for the eyes.

**Figure 11:**
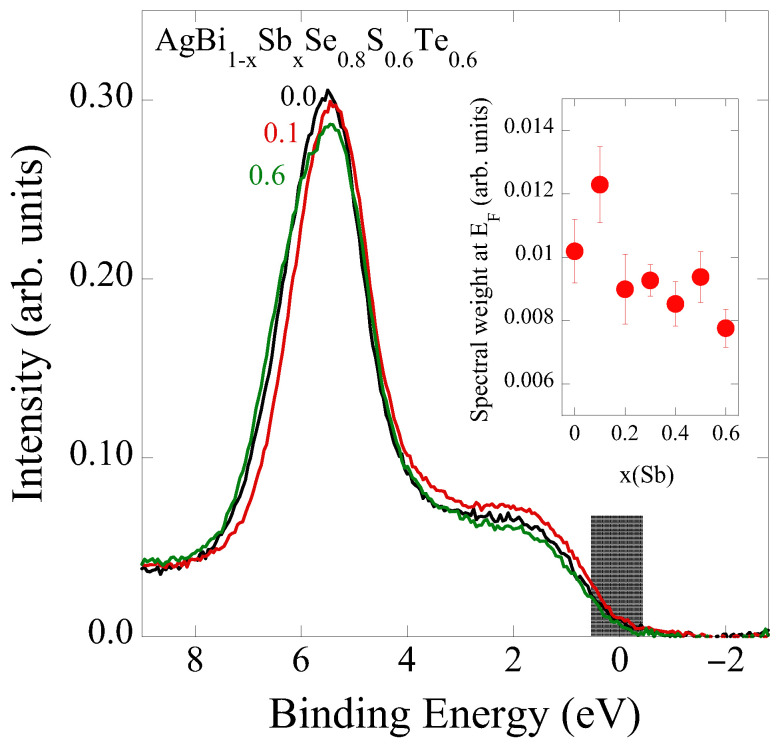
Valence bands of Bi_1−*x*_Sb_*x*_Se_0.8_S_0.6_Te_0.6_ for several Sb substitutions. The inset shows the spectral weight integrated around the Fermi level (shadowed window).

The line widths of different core levels are plotted in Figure 10. The Ag 3*d* core level tends to become sharper, which can indicate reduced configurational disorder around Ag. In light of the $\sigma^{2}$ of Ag-Te showing a decrease with that of Ag-Se remaining constant, the decreasing width of Ag 3*d* suggests a preferential environment containing Se and Te. On the other hand, the Bi 4*f* core level shows significant sharpening for the x = 0.1 sample before becoming broader. Unlike Ag, the environment around Bi atoms may be dominated by S, as indicated by EXAFS data, revealing an increase in the $\sigma^{2}$ of Bi-S. Similar behavior is observed for the Sb 3*d* core levels, which show systematic broadening with Sb substitution for Bi. Thus, Ag/Bi anti-site disorder in Bi_1−*x*_Sb_*x*_Se_0.8_S_0.6_Te_0.6_ appears to be suppressed by Sb substitution. However, Bi/Sb anti-site disorder shows a likely emergence. A similar effect was noted previously in related compounds [42]. Further information on this can be obtained from the valence band spectra, shown in Figure 11.

As previously reported [35], the valence band of AgBi_1−*x*_Sb_*x*_Se_0.8_S_0.6_Te_0.6_ is mainly composed of Ag 5*d* orbitals mixed with Ch *p* orbitals (the main peak feature at ∼6 eV), while the states near the Fermi level are of Bi 6*p*/Sb 5*p* character [43]. The integrated spectral intensity around E_*F*_ is shown in the inset. For small Sb substitution (x = 0), there is a sharp increase in the Fermi level population, possibly due to a donor level created by Sb substitution close to the conduction band. Further Sb substitution induces a small decrease in the Fermi level population, consistent with an overall increase in resistivity for larger Sb content. A similar scenario was also presented for Bi_2−2*x*_Sb_*x*_Se_3_ by P. Losťak et al. [42], where the slight introduction of Sb was found to increase the number of free carriers in the system, a behavior that was reversed with further Sb addition.

Here, it is worth summarizing the outcomes of the experimental findings in light of the thermoelectric properties of AgBi_1−*x*_Sb_*x*_Se_0.8_S_0.6_Te_0.6_. The EXAFS results show an overall contraction of near-neighbor bond lengths for x ≤ 0.4; however, beyond this, the main effect seems to be an increase in the configurational disorder. The XANES spectra reveal a systematic change in the local geometry, with much larger changes observed around Bi. The XPS results suggest that Ag is in the Ag^1+^ state, across Sb substitution along with the disorder surrounding it. In contrast, the Bi and Sb core-level XPS spectra show that both atoms are in a single valence state for small Sb substitution, but quickly turn to a mixed valence state with further substitution. Similarly, the density of states at the Fermi level follows a comparable trend, increasing with small Sb substitution and then gradually decreasing. Examining the thermoelectric properties [31], the ZT remains higher for small Sb substitution and decreases drastically with further substitution. Thus, one can argue that the effect of Sb is beneficial only at low substitution levels due to the electronic component, since the density of states, and hence the electrical conductivity, is higher, while the local disorder is hardly affected. At higher Sb substitution, the local disorder undergoes some changes that affect the thermal conductivity; however, the decreased density of states drastically reduces the ZT of the system. Therefore, the interplay between the electronic structure and local disorder affects the thermoelectric properties of the system, in which Sb substitution appears beneficial only at low levels.

## 4. Summary and Conclusions

In summary, we have analyzed the local structure and electronic properties of the high-entropy type AgBi_1−*x*_Sb_*x*_Se_0.8_S_0.6_Te_0.6_ system with varying Sb substitution. Local structure measurements show an overall contraction of near-neighbor bond distances from Ag and Bi across the substitution range, which is also consistent with an overall shift of the XANES features associated with the chalcogen bonds. XPS measurements, on the other hand, reveal a sharp change in the Ag and Bi core levels for small Sb substitution, followed by a systematic change. We find that Bi and Sb are in a mixed-valent state in Sb-substituted samples, except for those with a small substitution. The valence band shows an increase in the density of states at E_*F*_ with small Sb substitution, followed by a progressive decrease. This is consistent with the progressive increase in activation energy observed in the same materials, resulting in increased resistivity of AgBi_1−*x*_Sb_*x*_Se_0.8_S_0.6_Te_0.6_ with increasing Sb content. It appears that Ag/Bi anti-site disorder decreases; however, Bi/Sb anti-site disorder tends to increase with Sb substitution and seems to correlate with the mixed valence of Bi and Sb. In conclusion, the findings of this work provide a clear indication that small Sb substitution can be beneficial for the thermoelectric properties of AgBi_1−*x*_Sb_*x*_Se_0.8_S_0.6_Te_0.6_. The results also indicate that the interplay between local structure and electronic structure is a limiting factor for any further benefit from Sb substitution for Bi. Further insights can be obtained from temperature-dependent EXAFS measurements, which provide key information on bond length characteristics.

## Data Availability

The original contributions presented in the study are included in the article, further inquiries can be directed to the corresponding author.
